# Effects of cutting tool geometry on material removal of a gradient nanograined CoCrNi medium entropy alloy

**DOI:** 10.3762/bjnano.15.76

**Published:** 2024-07-23

**Authors:** Yu-Sheng Lu, Yu-Xuan Hung, Thi-Xuyen Bui, Te-Hua Fang

**Affiliations:** 1 Department of Mechanical Engineering, National Kaohsiung University of Science and Technology, Kaohsiung 807, Taiwan; 2 University of Technology and Education – The University of Danang, Danang, Vietnam

**Keywords:** CoCrNi, gradient nanograined materials, Hall–Petch, molecular dynamics, relative tool sharpness, removal mechanism

## Abstract

CoCrNi medium-entropy alloys (MEAs) have attracted extensive attention and research because of their superior mechanical properties, such as higher ductility, strength, and toughness. This study uses molecular dynamics (MD) simulations to investigate the cutting behavior of a gradient nanograined (GNG) CoCrNi MEA. Moreover, it explores the influence of relative tool sharpness and rake angle on the cutting process. The results show that an increase in the average grain size of the GNG samples leads to a decrease in the average resultant cutting force, as predicted by the Hall–Petch relationship. The deformation behavior shows that grain boundaries are crucial in inhibiting the propagation of strain and stress. As the average grain size of the GNG sample increases, the range of shear strain distribution and average von Mises stress decreases. Moreover, the cutting chips become thinner and longer. The subsurface damage is limited to a shallow layer at the surface. Since thermal energy is generated in the high grain boundary density, the temperature of the contact zone between the substrate and the cutting tool increases as the GNG size decreases. The cutting chips removed from the GNG CoCrNi MEA substrates will transform into a mixed structure of face-centered cubic and hexagonally close-packed phases. The sliding and twisting of grain boundaries and the merging of grains are essential mechanisms for polycrystalline deformation. Regarding the cutting parameters, the average resultant force, the material accumulation, and the chip volume increase significantly with the increase in cutting depth. In contrast to sharp tools, which mainly use shear deformation, blunt tools remove material by plowing, and the cutting force increases with the increase in cutting-edge radius and negative rake angle.

## Introduction

Compared with traditional alloys, high-entropy alloys (HEAs) with multiple elements exhibit diverse and unprecedented mechanical properties, attracting widespread scientific attention and research [1–2]. Among them, the ternary medium-entropy alloy (MEA) CoCrNi and its derived five-element CoCrFeMnNi HEA [3–4] have been found to exhibit high strength and ductility. Weng et al. used laser-aided additive manufacturing to fabricate a CoCrNi MEA with a perfect combination of strength and ductility [5]. Zhang et al. fabricated a new transformable FeCoCrNiMn HEA with both face-centered cubic (FCC) and body-centered cubic (BCC) phases to enhance the strength and ductility of the HEA [6]. This has been attributed to the low stacking-fault energy of this type of alloy. The structure of such substrates is more prone to generating twinning boundaries with hexagonally close-packed (HCP) phases during the deformation process, which enables better performance in low-temperature environments, such as industry in cold areas and aerospace. Ke et al. fabricated a high-quality MEA (NiCoCr)_95_V_5_, which achieved an excellent strength and plasticity product exceeding 86 GPa·% at low temperatures [7]. Qiu et al. investigated the effects of adding Al, Ti, Mo, and W on low-temperature phase stability, mechanical properties, and deformation behavior of CoCrNi-based MEAs [8].

Strengthening through grain refinement can increase the strength further without adjusting the composition. Wei et al. used a mechanical surface abrasion treatment to prepare a CoCrNi MEA with a grain-size-gradient structure with excellent strain hardening potential compared with spark plasma sintering fine-grained CoCrNi MEA [9]. Lu et al. revealed the mechanism of the excellent strength, ductility, and work-hardening capacity of gradient nanograined (GNG) CrCoNi MEA samples [10]. However, it is complicated to fabricate experimentally GNG samples and to examine gradient grain structures. Therefore, this study investigates the effects of GNG structures, relative tool sharpness (RTS), and rake angle on the cutting behavior of a CoCrNi MEA using molecular dynamics simulations.

## Methods

**Figure 1:**
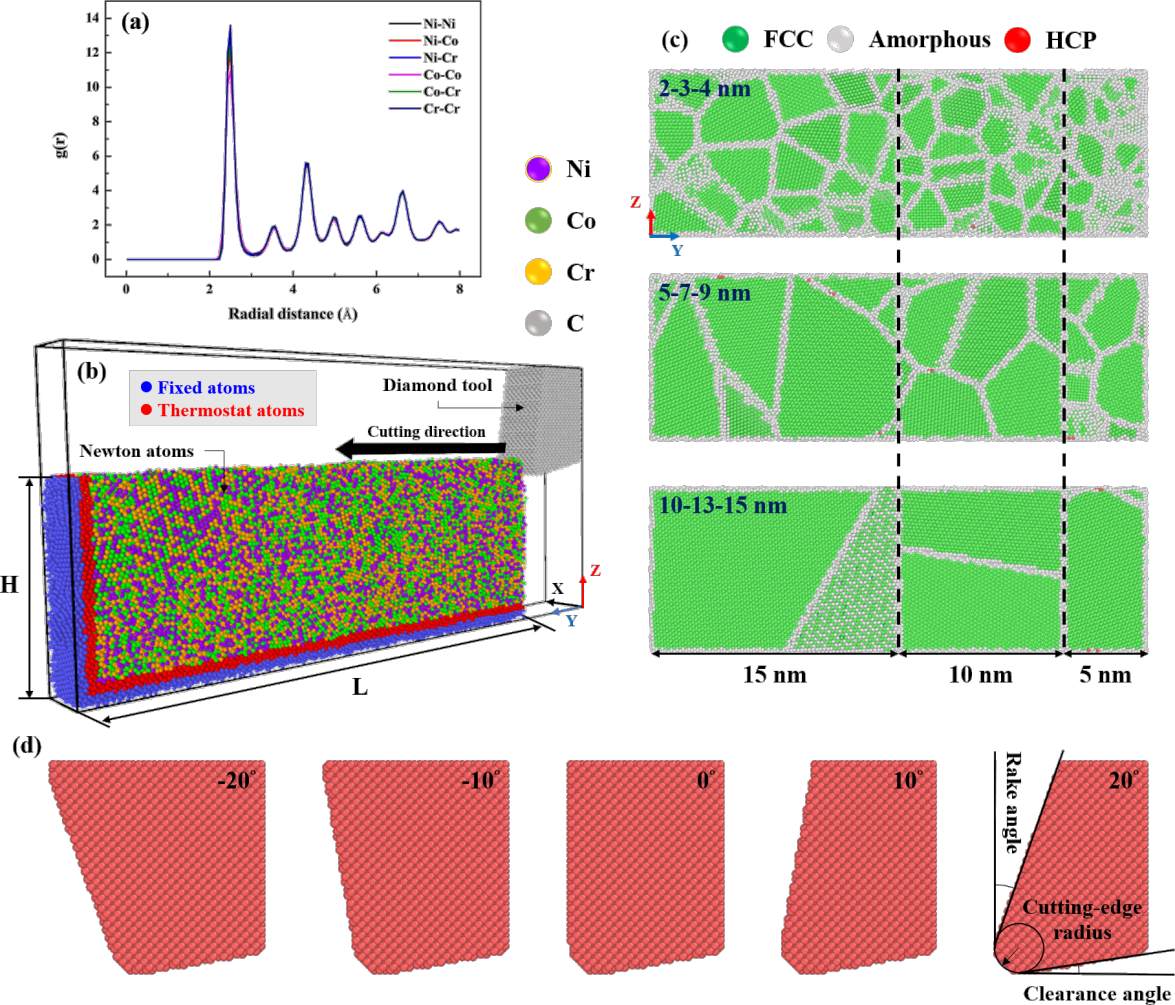
(a) Radial distribution function diagram, (b) physical model of the CoCrNi MEA substrate in the cutting process, (c) polycrystalline structure with the different gradients, and (d) cutting tool with various rake angles.

The cutting simulation model was established to explore the characteristics of plastic deformation and material removal of a GNG CoCrNi MEA, as shown in Figure 1. FCC phase GNG CoCrNi MEA substrates with dimensions of 3 nm × 30 nm × 10 nm were used for all cases in this study. The polycrystalline samples consist of randomly oriented grains. The MEA substrates contain 25,952 Co atoms, 26,628 Cr, and 26,746 Ni atoms. Hence, the alloy composition of the CoCrNi MEA is Ni_32.7_Co_33.6_Cr_33.7_. The radial distribution function, *g*(*r*), of the CoCrNi MEA substrates was calculated, proving the correctness of the structure, as shown in Figure 1a. Fixed atomic and thermostat layers were added to the CoCrNi MEA substrates to facilitate the machining simulation, as shown in Figure 1b. Figure 1c shows polycrystalline CoCrNi MEAs with different grain size gradient sizes, including 2-3-4 nm, 5-7-9 nm, and 10-13-15 nm. Figure 1d shows the simulated diamond cutting tool, a rigid body with various rake angles consisting of 14,287–39,156 C atoms. Periodic boundary conditions are placed on the *x* axis, while the remaining axes are non-periodic. The temperature is 300 K. The canonical ensemble (NVT) is used in the equilibration process, and the microcanonical ensemble (NVE) is used to consider the thermal change during the cutting process. The simulation was carried out with time steps of 0.001 ps and a 10 ps equilibrium process. Table 1 shows the MD simulation parameters of the GNG CoCrNi MEA cutting process for this study.

**Table 1:** MD simulation parameters in this study.

Parameter	Specimen	Tool

material	CoCrNi MEA	diamond
size (nm)	3 × 30 × 10	3 × 5 × 7
cutting-edge radius (nm)	—	0.5, 1, 2
rake angle (°)	—	20, 10, 0, −10, −20
clearance angle (°)	—	10
number of atoms	79,288	14,287–39,156
cutting depth (nm)	0.5, 1, 2	—
cutting rate (m/s)	10	—
temperature (K)	300	—
time step (ps)	0.001	—
potential	EAM + LJ	—
ensemble	NVT + NVE	—

The embedded atom method (EAM) potential from Farkas et al. [11] was utilized for modeling the atomic interaction of the atoms in the CoCrNi MEA substrates. The Lennard-Jones (LJ) potential was used to model the interaction among tool and sample atoms, and among the atoms in the tool [12–15]. The parameters of the LJ potential are displayed in Table 2.

**Table 2:** LJ potential parameters for CoCrNi MEA substrate and cutting tool.

Interaction	ε (eV)	σ (Å)

C–Co	0.124	2.703
C–Cr	0.123	2.718
C–Ni	0.125	2.691

The MD cutting simulations were carried out using the large-scale atomic/molecularly parallel simulator (LAMMPS) software [16–17], and the GNG model substrates were built from the ATOMSK software [18]. Deformation response, molecular structure, and atomic strain behavior were observed using the Open Visualization Tool (OVITO) software [19–20]. The mechanisms of phase transitions and partial dislocation motions in crystalline materials were examined by common neighbor analysis (CNA) of the modules in OVITO. Cutting force, shear strain distribution, von Mises stress analysis, crystal structure evolution, temperature distribution, and calculation of material wear rate were investigated regarding the cutting behavior of GNG CoCrNi MEAs.

## Results and Discussion

### Effect of gradient nanograined structures

To investigate the influence of GNG structures on the cutting process of the CoCrNi MEAs in detail, six grain size gradient samples were prepared, namely 2-3-4, 5-7-9, and 10-13-15 nm samples (from small to large grains in the cutting direction), and 4-3-2, 9-7-5, 15-13-10 nm samples (from large to small grains in the cutting direction). The CoCrNi MEA substrates were cut with a cutting length of 20 nm and 1 nm depth with a cutting speed of 10 m/s at 300 K. The cutting-edge radius was fixed at 1 nm to analyze the surface morphology, atomic-scale wear, shear strain distribution, temperature distribution, and crystal structure evolution during cutting.

**Figure 2:**
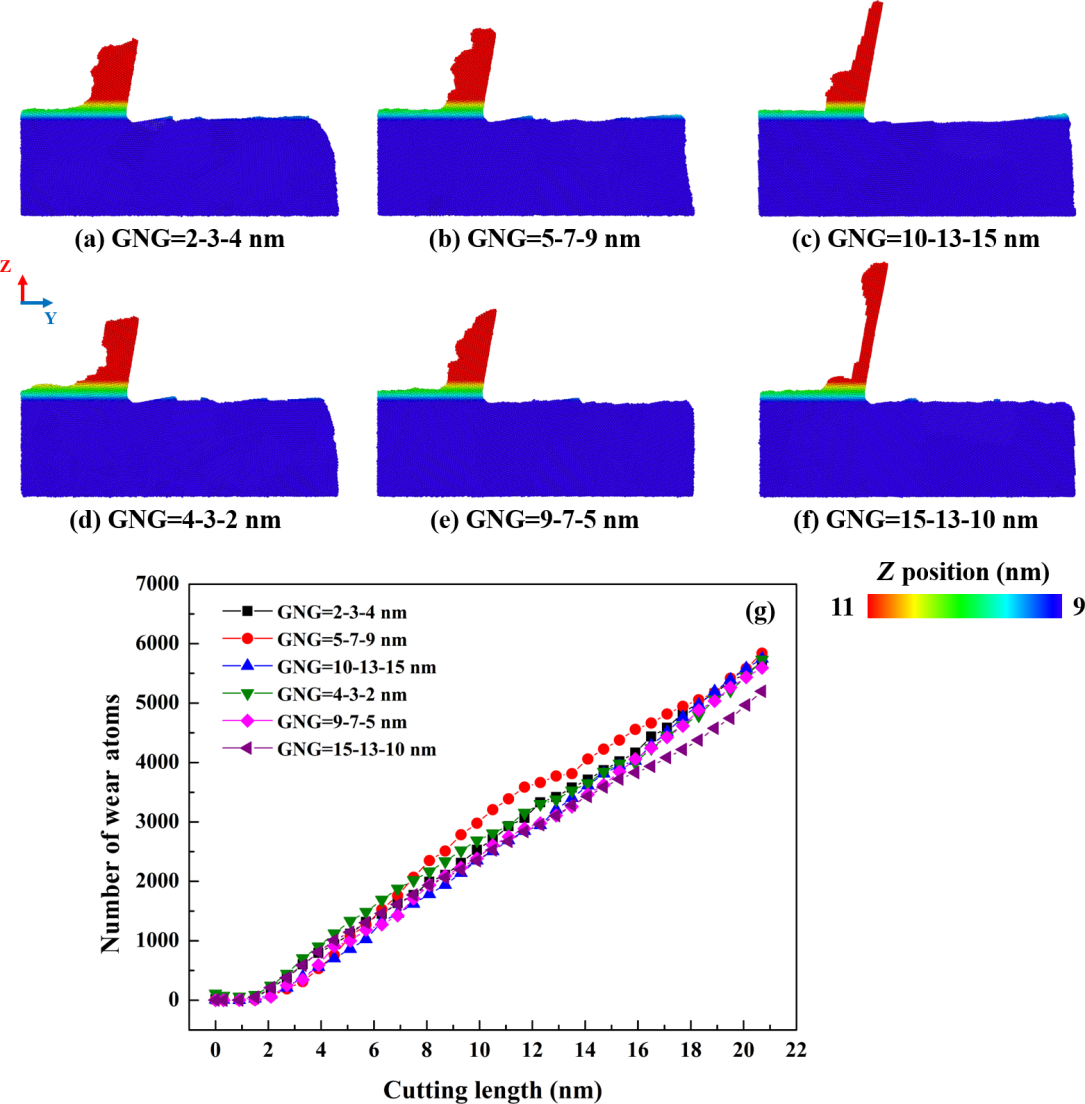
(a–f) Surface morphologies and (g) number of wear atoms for CoCrNi MEAs with various grain size gradients, with a tool rake angle of 10°, a tool cutting-edge radius of 1 nm, a cutting depth of 1 nm, a cutting speed of 10 m/s, and at a temperature of 300 K.

Figure 2a–f shows the atoms that pile-up on the surface of the CoCrNi MEA substrates. The number of wear atoms during cutting was calculated, as shown in Figure 2g. Grooves are formed as the material is removed by the tip of the cutting tool sliding across the substrate surface. Material removal is observed through the motion of atoms accumulated in front of the tool [21]. Furthermore, the atoms are unevenly distributed on both sides of the grooves. In general, in all cases, the pile-up height increases with increasing cutting length because much of the material accumulated around the tip of the cutting tool is squeezed upward during the cutting phase, resulting in a higher pile-up height. The results show that, when the cutting length increases, the number of wear atoms increases in all samples. The chips become elongated as the size of the grains increases. The sample with the grain size gradient of 15-13-10 nm exhibits the lowest number of wear atoms, while the remaining samples show similar numbers. The reason is that the material becomes softer in the presence of grain boundaries, which is consistent with previous literature [21,23]. The effects of different sliding systems on different grain sizes will affect the number of wear atoms in the GNG samples.

**Figure 3:**
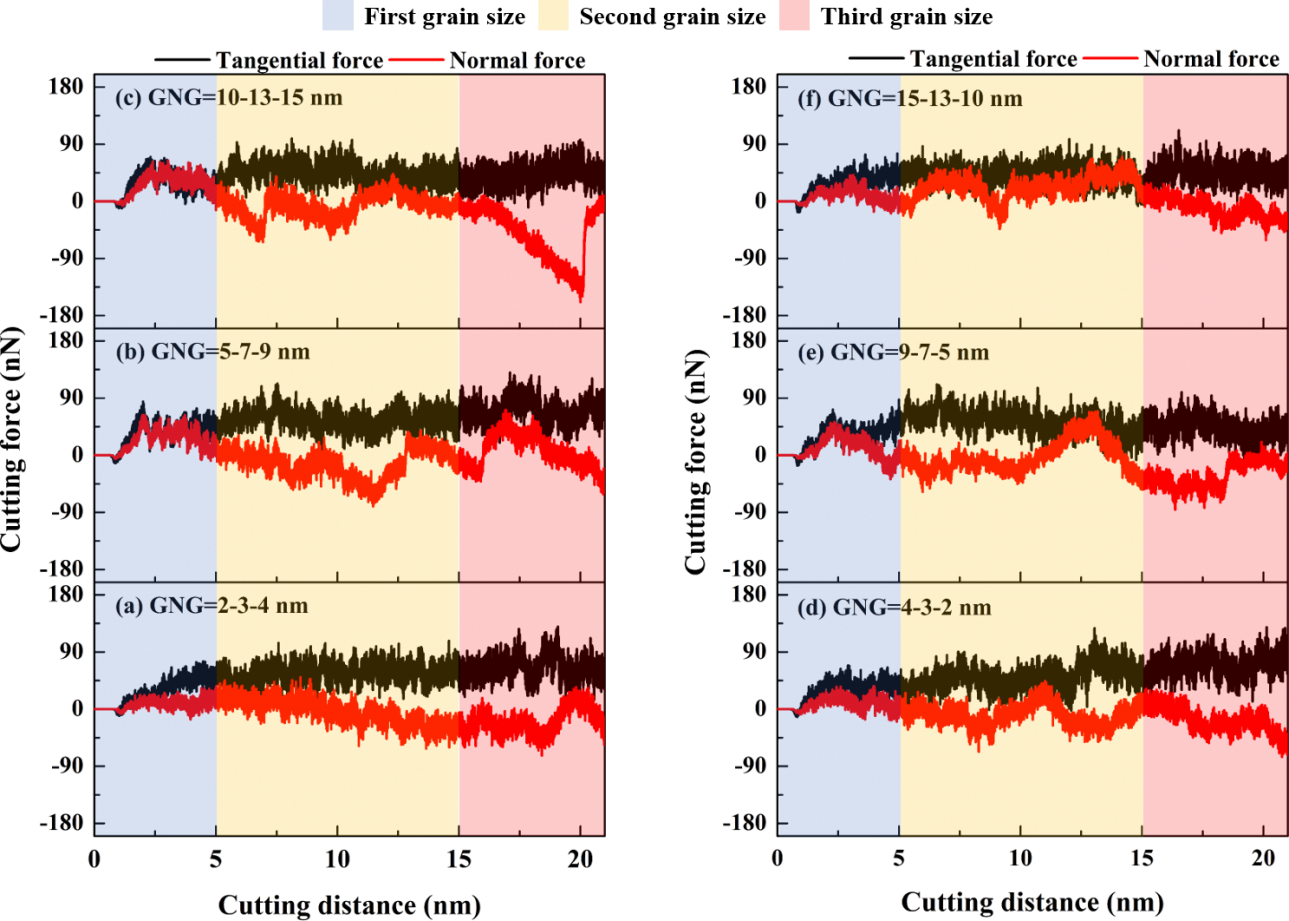
Force responses for CoCrNi MEAs with various grain size gradients of (a) 2-3-4 nm, (b) 5-7-9 nm, (c) 10-13-15 nm, (d) 4-3-2 nm, (e) 9-7-5 nm, and (f) 15-13-10 nm, with a tool rake angle of 10°, a tool cutting-edge radius of 1 nm, a cutting depth of 1 nm, a cutting speed of 10 m/s, and at a temperature of 300 K.

Figure 3 shows the relationship between the various GNG structures and the cutting force of GNG CoCrNi MEAs during the cutting process. In the cutting force response curve, the blue area represents the first grain size region, the yellow area represents the second, and the red area represents the third. The average resultant forces of the samples in Figure 3a–f are 56.4, 60.9, 51.5, 53.5, 51.8, and 45.1 nN, respectively. The results show that the sample in Figure 3b, with an average grain size of 6.31 nm, has the maximum average resultant force; the sample in Figure 3f, with an average grain size of 13.5 nm, has the smallest average resultant force. Bui et al. have shown that, as the grain size changes, the difference in friction coefficient with each cutting length is chaotic and does not follow the law of grain size increase [23]. The grain size affects the coefficient of friction in a rather complex way. Dislocation accumulation, lattice instability, and material accumulation may affect the cutting force response [21,24]. Furthermore, tangential forces are generated through work hardening resulting from the accumulation of chips or material pile-up [22]. The average resultant force is highest for the sample with a grain size gradient of 5-7-9 nm because, as the material pile-up increases, the contact area between the cutting tool and the specimen also increases. This leads to a rise in the average resultant force. Thicker chips result in higher cutting resistance strength. The sample with a grain size gradient of 15-13-10 nm with the thinnest chip thickness has the lowest average resultant force, consistent with the results of the surface morphology analysis in Figure 2.

**Figure 4:**
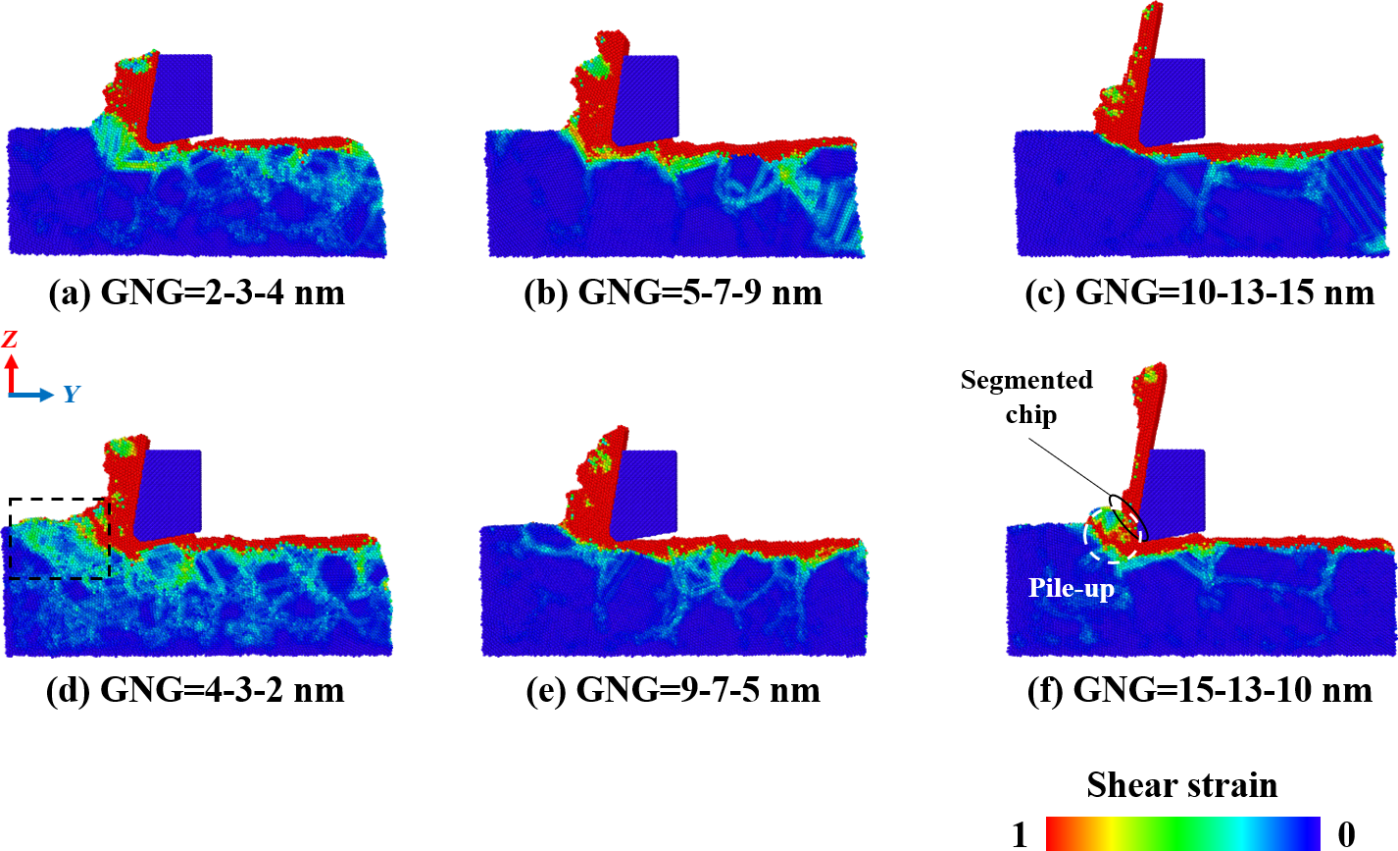
Shear strain distribution in CoCrNi MEAs with various grain size gradients of (a) 2-3-4 nm, (b) 5-7-9 nm, (c) 10-13-15 nm, (d) 4-3-2 nm, (e) 9-7-5 nm, and (f) 15-13-10 nm, with a tool rake angle of 10°, a tool cutting-edge radius of 1 nm, a cutting depth of 1 nm, a cutting speed of 10 m/s, and at a temperature of 300 K.

The subsurface quality of the workpiece significantly affects accuracy and durability in the machining process. To investigate the deformation mechanism of the subsurface, this study analyzed the influence of the cutting tool on the local stress and strain distribution in the CoCrNi MEA substrates [25]. Figure 4 shows the shear strain distribution in CoCrNi MEA samples with various grain size gradients during the cutting process. The results reveal that shear strain mainly propagates through grain boundaries. For samples with a small GNG, the smaller the grain size, the greater the grain boundary density and the wider the shear strain diffusion range into the material’s interior [26]. As the average grain size of the GNG sample increases, the range of shear strain influence decreases, and the internal strain gradually converges within the grain boundaries and in front of the tool. This is because the density of the grain boundaries decreases and is affected by deformation along the grain boundaries. There is also a corresponding reduction in the number of highly strained atoms that diffuse inward. As the average grain size increases, the chips gradually become thinner and longer. The subsurface damage in front of and below the tool is limited to a shallow layer at the surface.

The large grains of the samples with a large-to-small grain size gradient are squeezed by the tool and push the atoms of the smaller grains behind to move. Moreover, the shear strain of the sample in Figure 4d even extends to the uncut part of the substrate, which can explain why the cutting force of this sample increases in the later stages of cutting. The sample in Figure 4e exhibits a large cutting resistance at the early cutting stage. As the cutting length approaches the grain size region of 5 nm, the plastic deformation in front of the tool gradually turns into upward growth of chips, which also explains why the tangential force decreases. The sample in Figure 4f yields a segmented chip when cutting to a region close to the grain size of 10 nm, and a piece of the chip is removed. This sample exhibits minimal subsurface damage.

**Figure 5:**
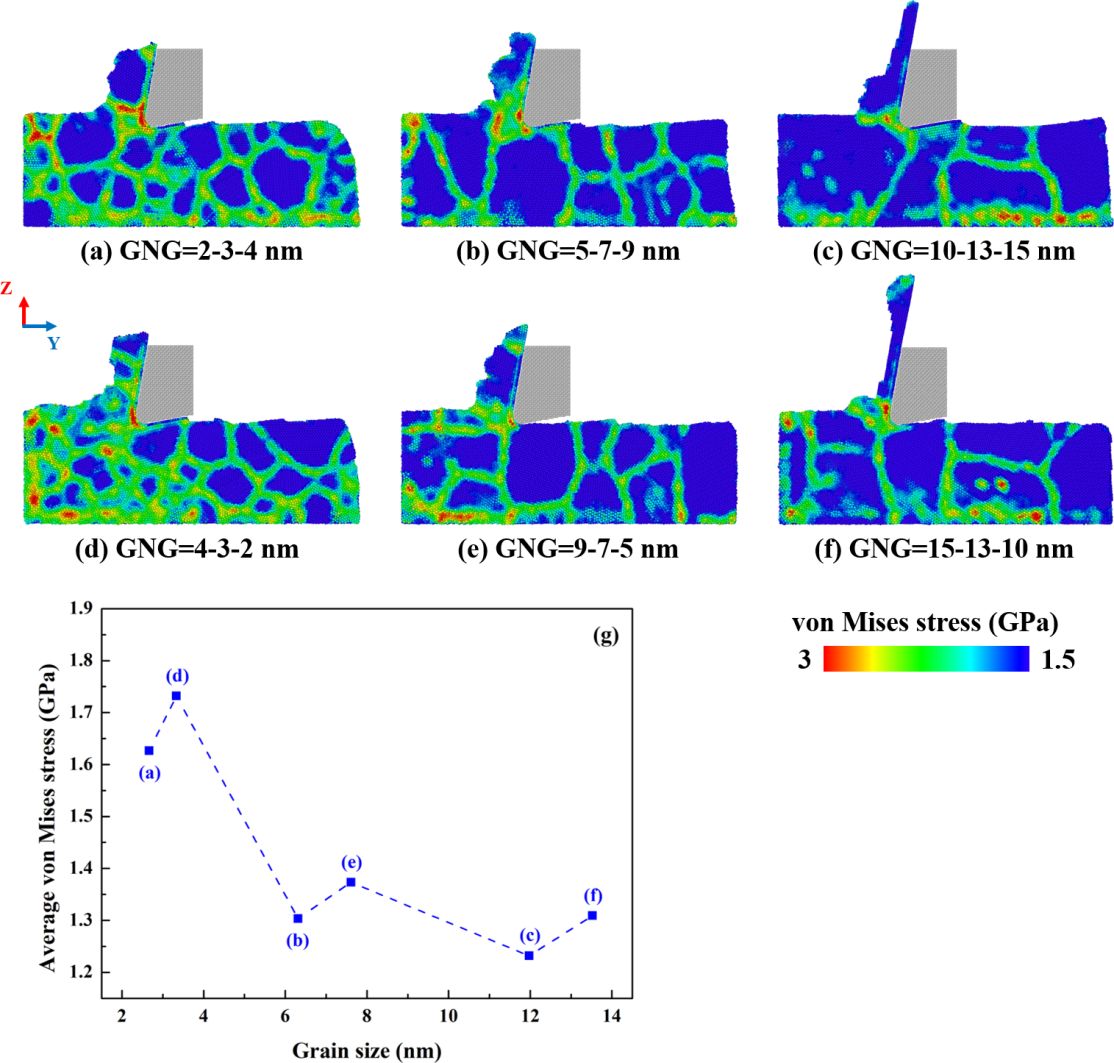
(a–f) Von Mises stress distribution in CoCrNi MEAs with various grain size gradients at a cutting length of 20 nm and (g) relationship between average von Mises stress and average grain size, with a tool rake angle of 10°, a tool cutting-edge radius of 1 nm, a cutting depth of 1 nm, a cutting speed of 10 m/s, and at a temperature of 300 K.

Figure 5a–f shows the von Mises stress distribution in CoCrNi MEA samples with various grain size gradients at a cutting length of 20 nm. The range of von Mises stress values from 1.5 to 3 GPa is depicted by the atoms’ color. Figure 5g displays the relationship between average von Mises stress and average grain size of GNG CoCrNi MEAs. The local distribution of von Mises stress was calculated through the following equation [27]:


[1] $$\sigma_{\text{vM}}={\left\{\frac{1}{2}\left[\begin{array}{l} {\left(\sigma_{xx}-\sigma_{yy}\right)}^{2}\\ +{\left(\sigma_{xx}-\sigma_{zz}\right)}^{2}\\ +{\left(\sigma_{zz}-\sigma_{yy}\right)}^{2} \end{array}\right]+3\left(\sigma_{xy}^{2}+\sigma_{xz}^{2}+\sigma_{yz}^{2}\right)\right\}}^{1/2}.$$

The results show a relatively concentrated stress distribution at the tip of the cutting tool. In polycrystalline materials, local stress is mainly concentrated at or near grain boundaries with amorphous structures, creating a complex stress distribution for the expansion of the amorphous regions [28–29]. The local stress on the grain boundaries of the sample forms a clear network of contour maps [30]. The anisotropy of the grains causes anisotropic deformation and increases the surface dispersion density. Therefore, the stress required to promote plastic deformation rises more at grain boundaries than inside grains [23,31]. The number of grain boundaries increases with decreasing average grain size, resulting in a more significant increase in local stress in the cutting zone and at the grain boundaries of the matrix [32]. Moreover, the average von Mises stress decreases as the average grain size increases. In summary, grain boundaries play an essential role in the propagation of deformation from the cutting zone to the substrate.

**Figure 6:**
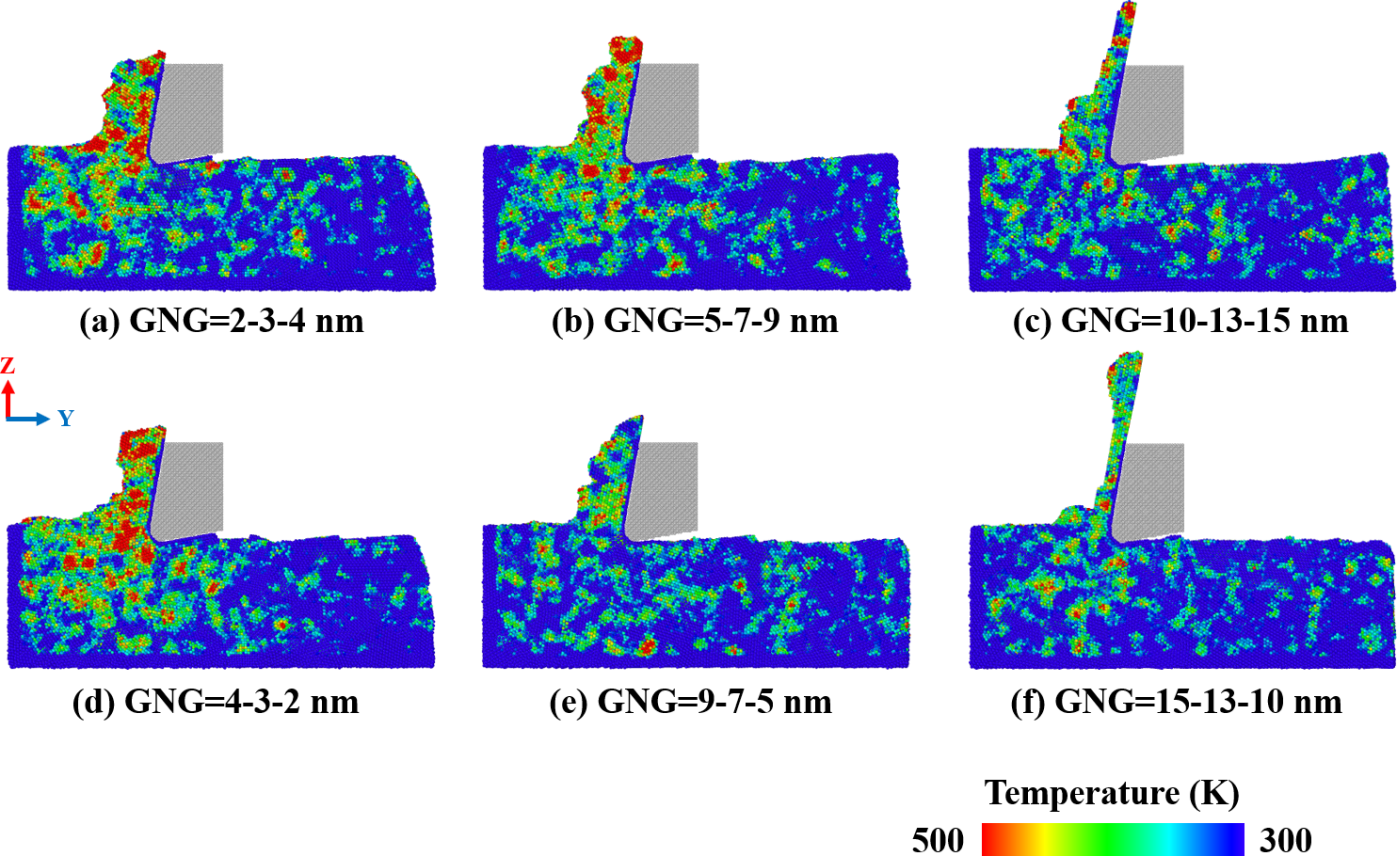
Temperature distribution in CoCrNi MEAs with various grain size gradients of (a) 2-3-4 nm, (b) 5-7-9 nm, (c) 10-13-15 nm, (d) 4-3-2 nm, (e) 9-7-5 nm, and (f) 15-13-10 nm, with a tool rake angle of 10°, a tool cutting-edge radius of 1 nm, a cutting depth of 1 nm, a cutting speed of 10 m/s, and at a temperature of 300 K.

Figure 6 shows the temperature distribution in CoCrNi MEA samples with various grain size gradients during the cutting process. The temperature distribution was obtained according to the relationship between temperature and energy [33]:


[2] $$T=\frac{\sum_{j=1}^{N}m_{j}v_{j}^{2}}{3k_{\text{B}}N_{\text{atoms}}}.$$

where *k*_B_, *N*_atoms_, and *m_j_* are the Boltzmann constant, the number of atoms of particles in each cell, and the mass of the *j*-th atom, respectively. The temperature of atoms is indicated by the atom color, with red indicating the highest temperature. The temperature is proportional to the kinetic energy, so the system releases much kinetic energy when the deformation process changes from elastic to plastic deformation. The kinetic energy of the atoms in the contact area between the cutting tool and the substrate is the highest, leading to the highest temperature at this position. Samples with small grain gradients, corresponding to a higher number of amorphous atoms, exhibit an increase in mixture disorder of the atoms, leading to higher kinetic energy, which generates more thermal energy [34].

**Figure 7:**
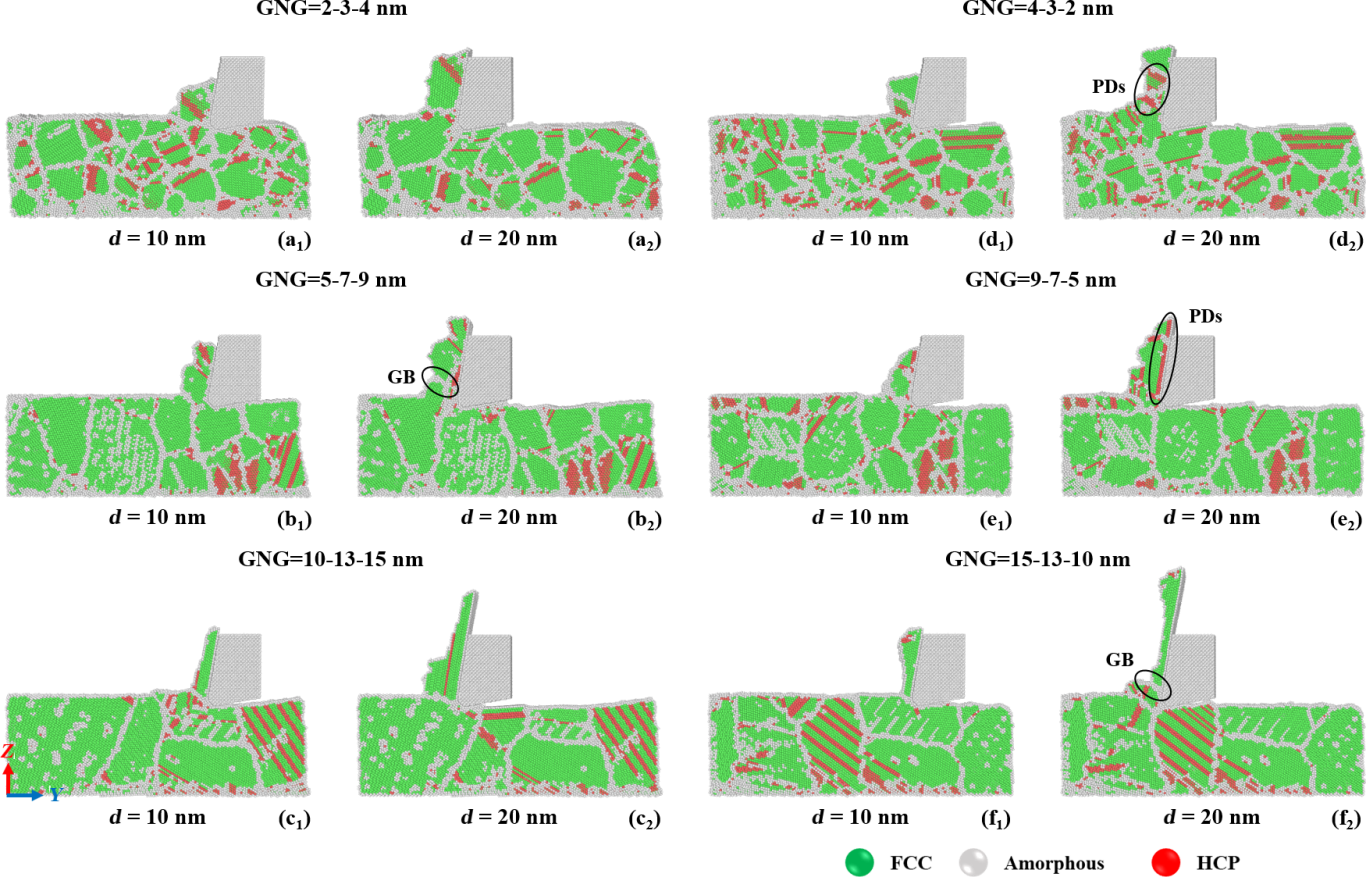
Crystal structure evolution in CoCrNi MEAs with various grain size gradients of (a) 2-3-4 nm, (b) 5-7-9 nm, (c) 10-13-15 nm, (d) 4-3-2 nm, (e) 9-7-5 nm, and (f) 15-13-10 nm, with a tool rake angle of 10°, a tool cutting-edge radius of 1 nm, a cutting depth of 1 nm, a cutting speed of 10 m/s, and at a temperature of 300 K.

Figure 7 displays the crystal structure evolution in CoCrNi MEAs with various grain size gradients during the cutting process. CNA was used to identify the structural phase transformation and amorphization processes of CoCrNi MEAs during cutting. The subsurface damage mechanism was investigated through the structural transformation of surface atoms [35–36]. The subsurface damage increases as the cutting length increases. The chips removed during machining of nanocrystalline materials are mainly amorphous structures. In this study, the chips removed during the cutting process will transform into mixed-phase structures of FCC and HCP. The HCP phase usually accompanies partial dislocations such as twin boundaries and stacking faults. The results show that the plastic deformation of GNG CoCrNi MEAs with small grain sizes is mainly dominated by grain boundary slip and grain rotation [37–38]. The grain boundary energy is higher between grains with bigger size differences. The grains release energy as the machining proceeds and form larger grains, and the grain boundaries pin stacking faults and dislocations. When the grain size is large enough, partial dislocations such as stacking faults and twin boundaries are less susceptible to grain boundary interference. Therefore, only dislocation slip and twin boundary formation occur in grains of the sample in Figure 7c. The contact region between the chip and the cutting tool in the sample in Figure 7b has denser grain boundaries and stacking fault extrusions, which also explains why the von Mises stress in this sample is more diffuse, as shown in Figure 5. For samples with large-to-small grain size gradients, as the grain size decreases, the grain boundaries gradually prevent dislocation sliding, and more stacking faults and twins occur. Stacking faults and grain boundaries are also inside the chips of the samples in Figure 7d and Figure 7e.

### Effect of relative tool sharpness

Relative tool sharpness is the most critical parameter in ultraprecision machining [39]. It is defined as the cutting depth (i.e., undeformed chip thickness, *a*) divided by the cutting-edge radius, *r* [40]. The RTS has been the subject of theoretical analyses, such as MD simulations [41], to predict the outcomes of ultraprecision machining [42]. This section investigates the effect of three RTS values of 0.5, 1.0, and 2.0 under various cutting depths (*a* = 0.5, 1.0, and 2.0 nm) and cutting-edge radii (*r* = 0.5, 1.0, and 2.0 nm) on the cutting process of GNG CoCrNi MEAs. The considered RTS parameters are summarized in Table 3.

**Table 3:** Considered parameters of relative tool sharpness simulations.

Cutting depth, *a* (nm)	Cutting-edge radius, *r* (nm)	RTS, *a*/*r*

0.5	1.0	0.5
1.0	2.0	0.5
1.0	1.0	1.0
1.0	0.5	2.0
2.0	1.0	2.0

### Cutting depth

**Figure 8:**
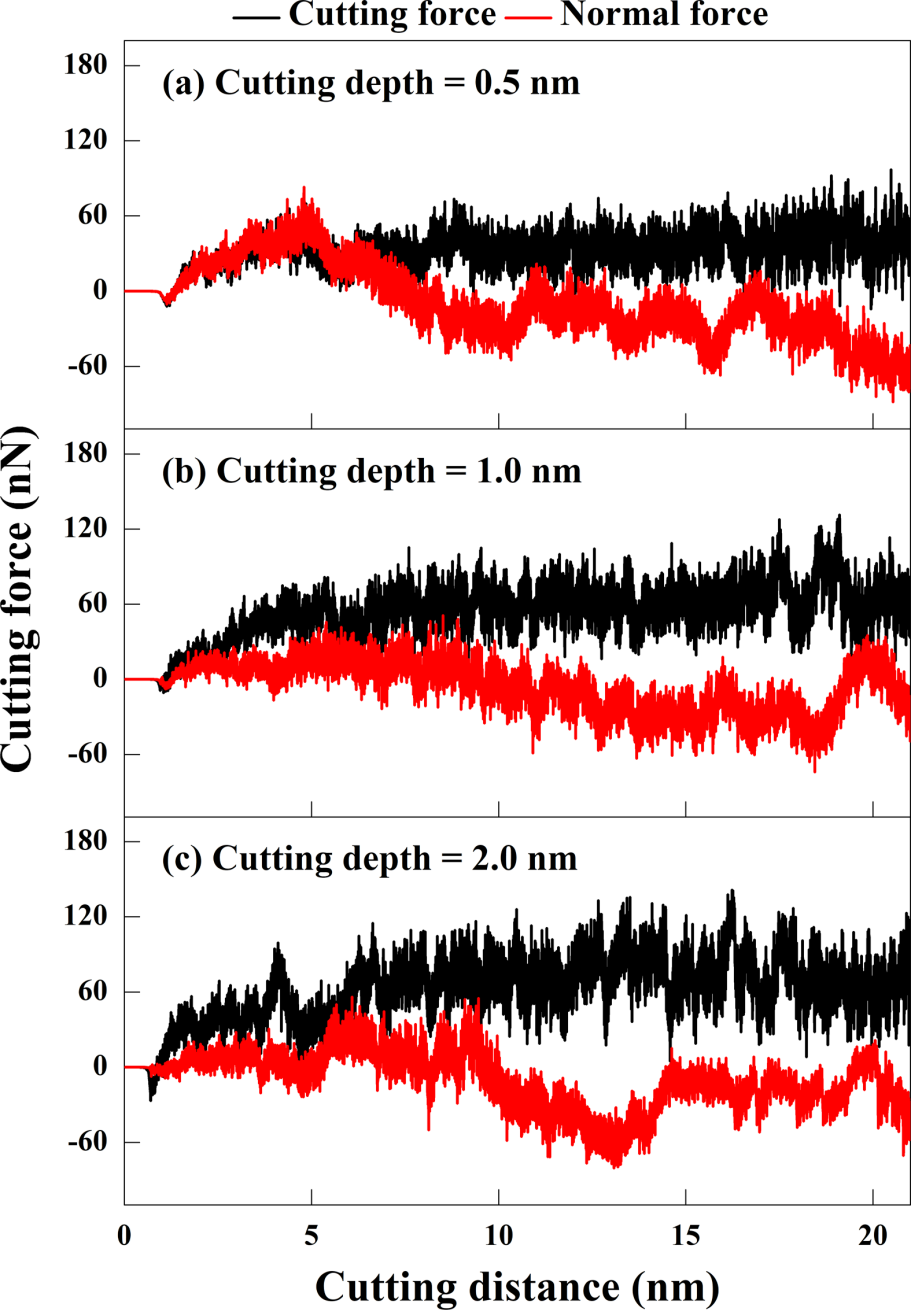
Force responses for GNG CoCrNi MEAs for various cutting depths of (a) 0.5 nm, (b) 1 nm, and (c) 2 nm, with a tool rake angle of 10°, a tool cutting-edge radius of 1 nm, a cutting speed of 10 m/s, and at a temperature of 300 K.

In conventional cutting, the cutting force increases with the cutting depth. When cutting at the nanoscale, cutting depth and cutting-edge radius are closely related to the cutting force [39]. Figure 8 shows the relationship between the cutting depth and the cutting force of GNG CoCrNi MEAs. The results show that, as the cutting depth increases, the amount of material removed at the front side of the cutting tool increases; hence, the change in cutting force is obvious. However, it is also affected by chip shape and defects such as grain boundaries and dislocations. The cutting force does not increase significantly at a cutting depth of 0.5 nm. Only as the cutting length increases, the number of accumulate atoms in the grooves slightly increases. When the cutting depth is 1.0 nm, the cutting force increases with increasing chip and grain sizes, and the oscillation amplitude of the cutting force tends to become larger, which is consistent with conventional cutting. For the cutting depth of 2.0 nm, the cutting force further increases. Chips accumulate in large quantities at the tool’s front end, and the cutting force even decreases slightly at later stages of the cutting. It may be that the chips affect the cutting mechanism such that the cutting force’s oscillation amplitude is the highest.

During the cutting process, the tool will sink because of the compressive force generated by the upward accumulation of chips on the front side of the tool. Still, the frictional sliding during the chip accumulation will suspend the normal force increase. For a cutting depth of 0.5 nm, the contact area between the material surface and the tool is small, and only a few atoms accumulate at the tip of the cutting tool. The subsurface atoms push the tool upward until the chips increase at a cutting length of about 5 nm, and the normal force changes. The average resultant forces for cutting depths 0.5, 1.0, and 2.0 nm are 32.8, 53.0, and 61.6 nN, respectively, indicating that the average resultant force increases significantly with the increase in cutting depth.

**Figure 9:**
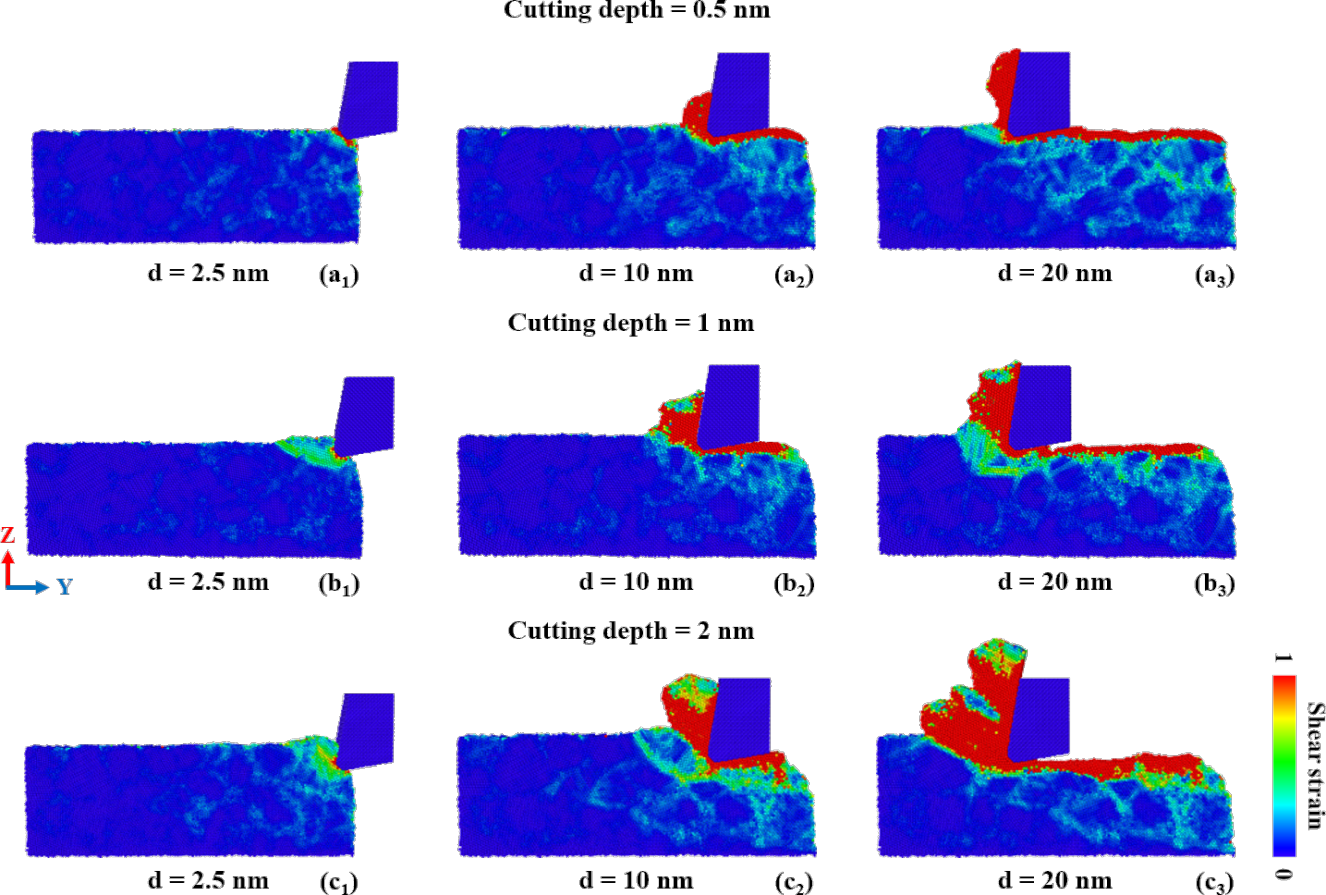
Shear strain distribution in GNG CoCrNi MEAs for various cutting depths of (a) 0.5 nm, (b) 1 nm, and (c) 2 nm, with a tool rake angle of 10°, a tool cutting-edge radius of 1 nm, a cutting speed of 10 m/s, and at a temperature of 300 K.

Figure 9 compares the shear strain distribution in GNG CoCrNi MEAs for various cutting depths during the cutting process. The results show that, as the cutting depth increases, the number of atoms under high shear strain generated by the plastic deformation increases significantly, and the strain spreads wider through the grain boundaries into the interior of the substrate. The shear strain generated by the accumulated atoms at the tip of the tool and the subsurface damage of the substrate is relatively large, leading to atomic displacement and plastic deformation. As the cutting depth becomes shallower, the generated chips are smaller, and the shear strain propagation range is smaller. As the cutting depth increases, chips and accumulated atoms on the contact surface between the tool and the substrate generate many shear bands and squeeze the grains behind the substrate. Therefore, plastic deformation and subsurface damage become more significant with a deeper cutting depth.

**Figure 10:**
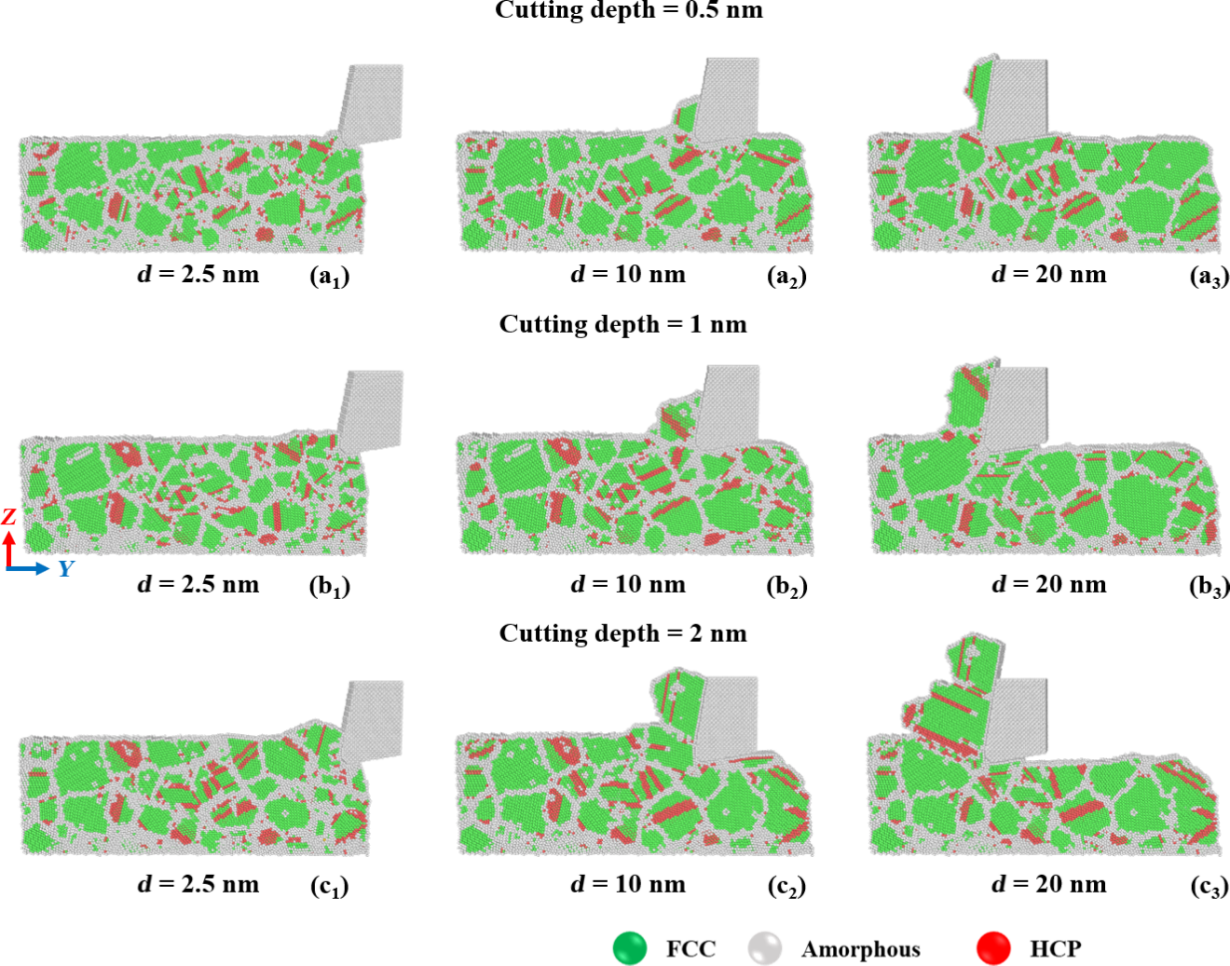
Crystal structure evolution in GNG CoCrNi MEAs for various tool cutting depths of (a) 0.5 nm, (b) 1 nm, and (c) 2 nm, with a tool rake angle of 10°, a tool cutting-edge radius of 1 nm, a cutting speed of 10 m/s, and at a temperature of 300 K.

Figure 10 displays the crystal structure evolution in GNG CoCrNi MEAs for various cutting depths during the cutting process. The results show that the crystal structure changes from the FCC phase to the HCP phase during cutting. Most grain boundaries and stacking faults re-transform into twin boundaries or reorganize into larger grains as the cutting proceeds. For the sample with a cutting depth of 2.0 nm, there is an apparent dislocation slip where the chips accumulate, which explains the significant accumulation of chip atoms at the tip of the tool.

### Cutting-edge radius

**Figure 11:**
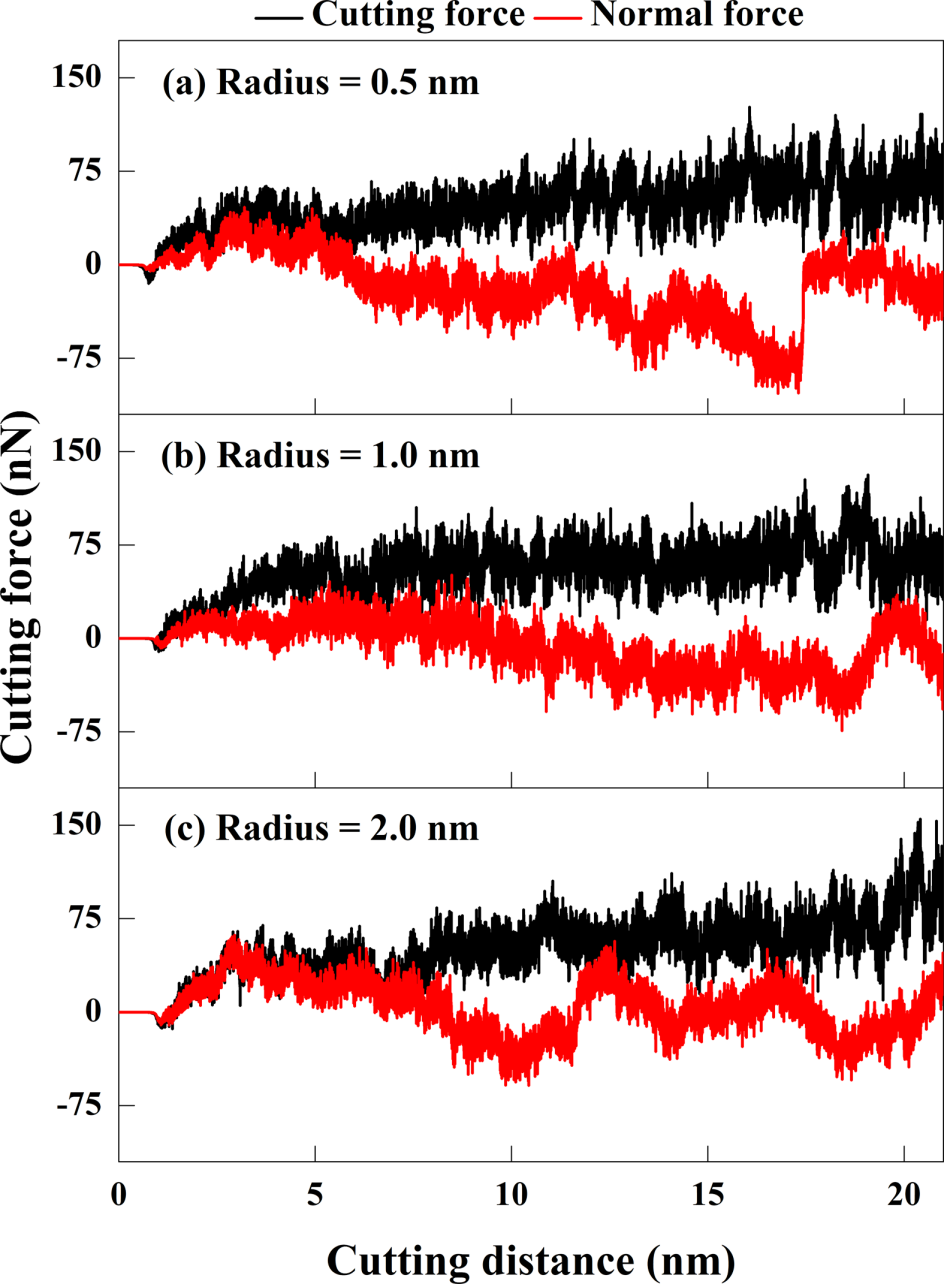
Force responses for GNG CoCrNi MEAs for various tool cutting-edge radii of (a) 0.5 nm, (b) 1 nm, and (c) 2 nm, with a tool rake angle of 10°, a tool cutting depth of 1 nm, a cutting speed of 10 m/s, and at a temperature of 300 K.

Figure 11 shows the relationship between the cutting-edge radius and the cutting force during the cutting process. The RTS increases as the cutting-edge radius decreases when the cutting depth is fixed. When the cutting-edge radius is 0.5 nm, the tool is the sharpest and has the best material removal rate. The blunt tool with a cutting-edge radius of 2.0 nm will preferably lead to plowing and squeezing for material removal at the initial cutting stage. It will switch to squeezing and shearing at later cutting stages. The results show that the cutting force increases with the blunter tip. When the RTS value is fixed, the tool nose radius is inversely proportional to the cutting depth, thus, affecting material removal rate and chip accumulation [39]. Therefore, the cutting force depends on the cutting-edge radius given equal cutting depths. The cutting tool generates extrusion and material accumulation when it first contacts the substrate. The cutting force increases slightly because of elastic recovery of the subsurface and chip generation and subsequently decreases because of the compression force generated by atomic accumulation. The sample with a cutting-edge radius of 0.5 nm produces chip breakage at a cutting length of about 16–17 nm. The cutting tool is then moved upward from the subsurface, and the normal force recovers to the value of the initial cutting stage. For the sample with a cutting-edge radius of 2.0 nm, the substrate is pushed with chip extrusion and friction, hence the oscillation of the normal force.

**Figure 12:**
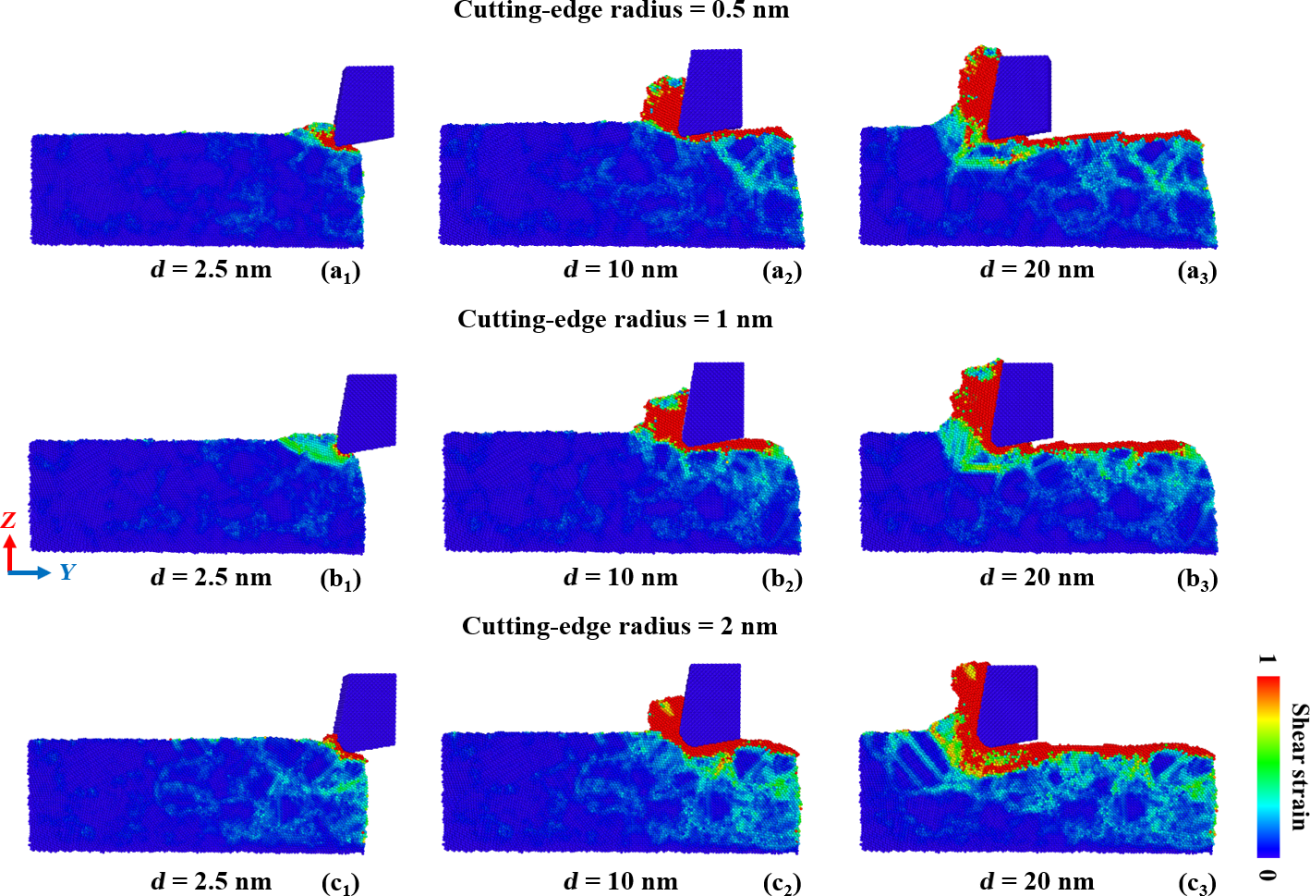
Shear strain distribution in GNG CoCrNi MEAs for various tool cutting-edge radii of (a) 0.5 nm, (b) 1 nm, and (c) 2 nm, with a tool rake angle of 10°, a cutting depth of 1 nm, a cutting speed of 10 m/s, and at a temperature of 300 K.

Figure 12 compares the shear strain distribution in GNG CoCrNi MEAs for various cutting-edge radii during the cutting process. The results show that the deformation mechanism of the chip from a cutting tool with a small cutting-edge radius is preferably shear deformation, and the shear strain is mainly distributed at the contact between the tool and substrate. As the cutting-edge radius increases, the subsurface damage range becomes larger, especially for samples with a cutting-edge radius of 2.0 nm. The complete mechanism, from plowing and squeezing to shearing, allows the plastic deformation range to extend beyond the front side and the bottom of the cutting tool, and significant shear strain diffusion appears even inside the substrate surface, which is deformed by the cutting trajectory.

**Figure 13:**
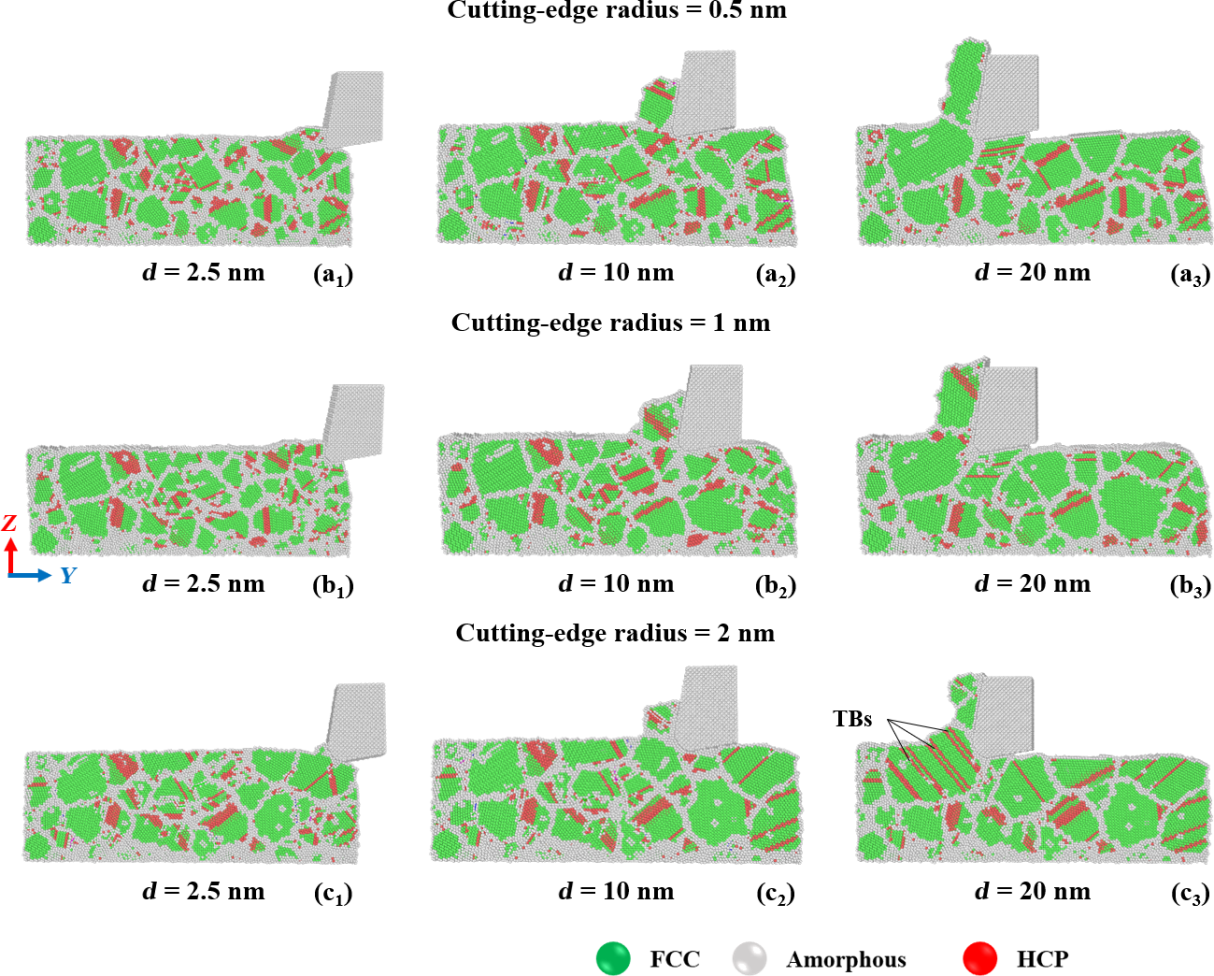
Crystal structure evolution in CoCrNi MEAs for various tool cutting-edge radii of (a) 0.5 nm, (b) 1 nm, and (c) 2 nm, with a tool rake angle of 10°, a cutting depth of 1 nm, a cutting speed of 10 m/s, and at a temperature of 300 K.

Figure 13 displays the crystal structure evolution in GNG CoCrNi MEAs for various cutting-edge radii during the cutting process. Again, the crystal structure of the CoCrNi MEA substrates changes from the FCC phase to the HCP phase during cutting. The results show that when the cutting-edge radius is 0.5 nm, the chips produce stacking faults and dislocation slips between grain boundaries, which decrease as the cutting length increases. Eventually, the grains around the chip reorganize into larger grains. For a cutting-edge radius of 2.0 nm, grains grow at the initial stages of cutting, and the rear grains are squeezed to cause slip and generate stacking faults and twin boundaries.

### Effect of rake angle

In conventional cutting, a positive rake angle makes the tool sharper, reducing cutting and normal forces. It helps form continuous chips in ductile materials and avoids the retention of accumulated chips. Generating a negative rake angle blunts the tool and increases the cutting edge’s strength and cutting force. Therefore, this section will investigate the influence of different rake angles on the cutting process of GNG CoCrNi MEAs.

**Figure 14:**
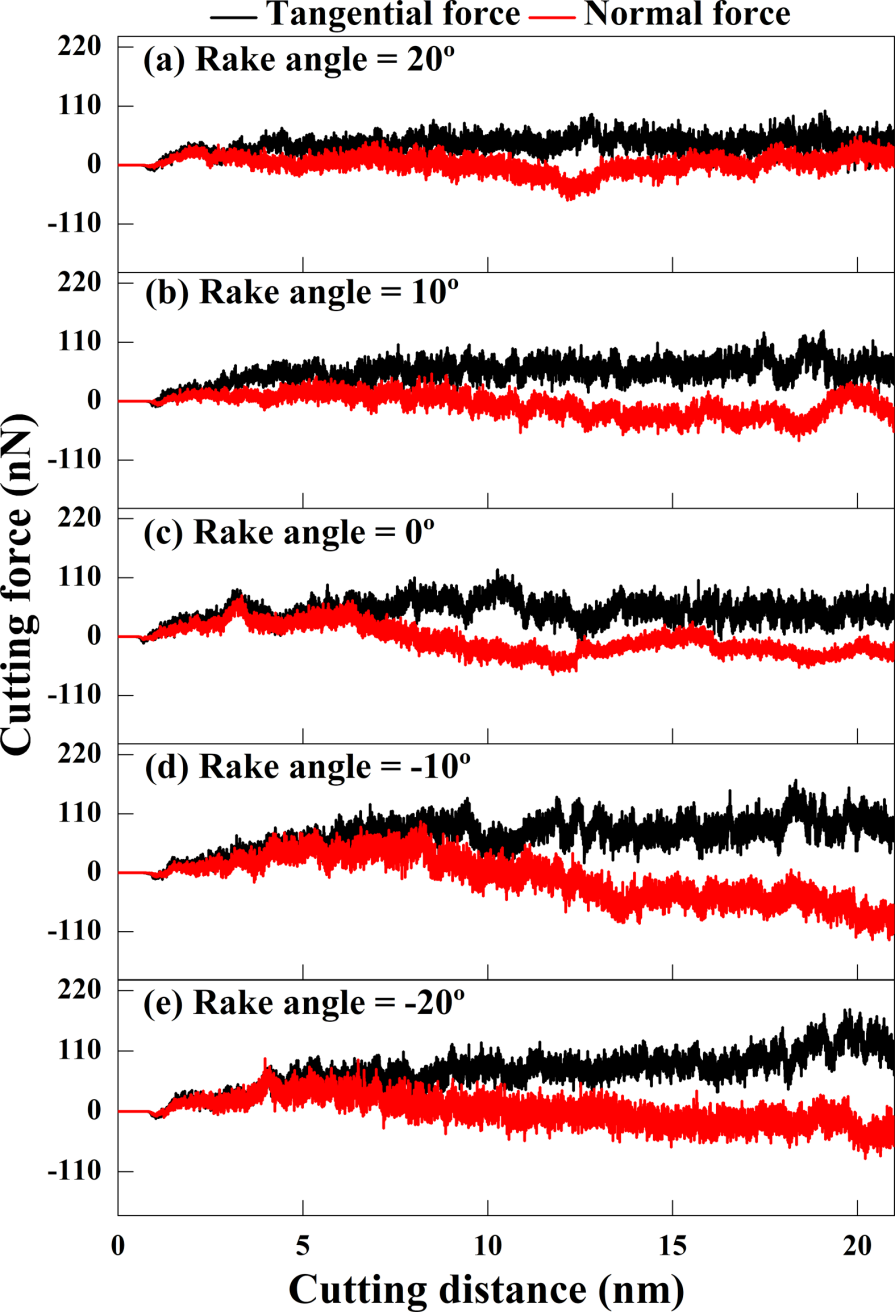
Force responses for GNG CoCrNi MEAs for various tool rake angles of (a) 20°, (b) 10°, (c) 0°, (d) −10°, and (e) −20°, with a cutting depth of 1 nm, a tool cutting-edge radius of 1 nm, a cutting speed of 10 m/s, and at a temperature of 300 K.

Figure 14 shows the relationship between the rake angle and the cutting force of GNG CoCrNi MEAs during the cutting process. The results show that the cutting force is smaller at sharper positive rake angles. As the rake angle decreases (i.e., the tool becomes more blunt), the cutting force gradually increases. The cutting process is mainly dominated by shearing. When the tool exhibits a negative rake angle, it gradually becomes shearing driven by atomic extrusion. Stronger accumulation of atoms and deformation occur in front of the tool, and the atoms will slide along the tangential direction after being attached to the tool. Because of the downward compressive stress generated by the accumulation of chips in front of the tool, the normal force increases and is accompanied by more significant oscillations. This also means that using a tool with a negative rake angle will make the machining process less stable. There are relatively similar force trajectories for rake angles of 0° and −10°. When the rake angle is negative, the horizontal cutting force increases as the thrust force decreases [43].

**Figure 15:**
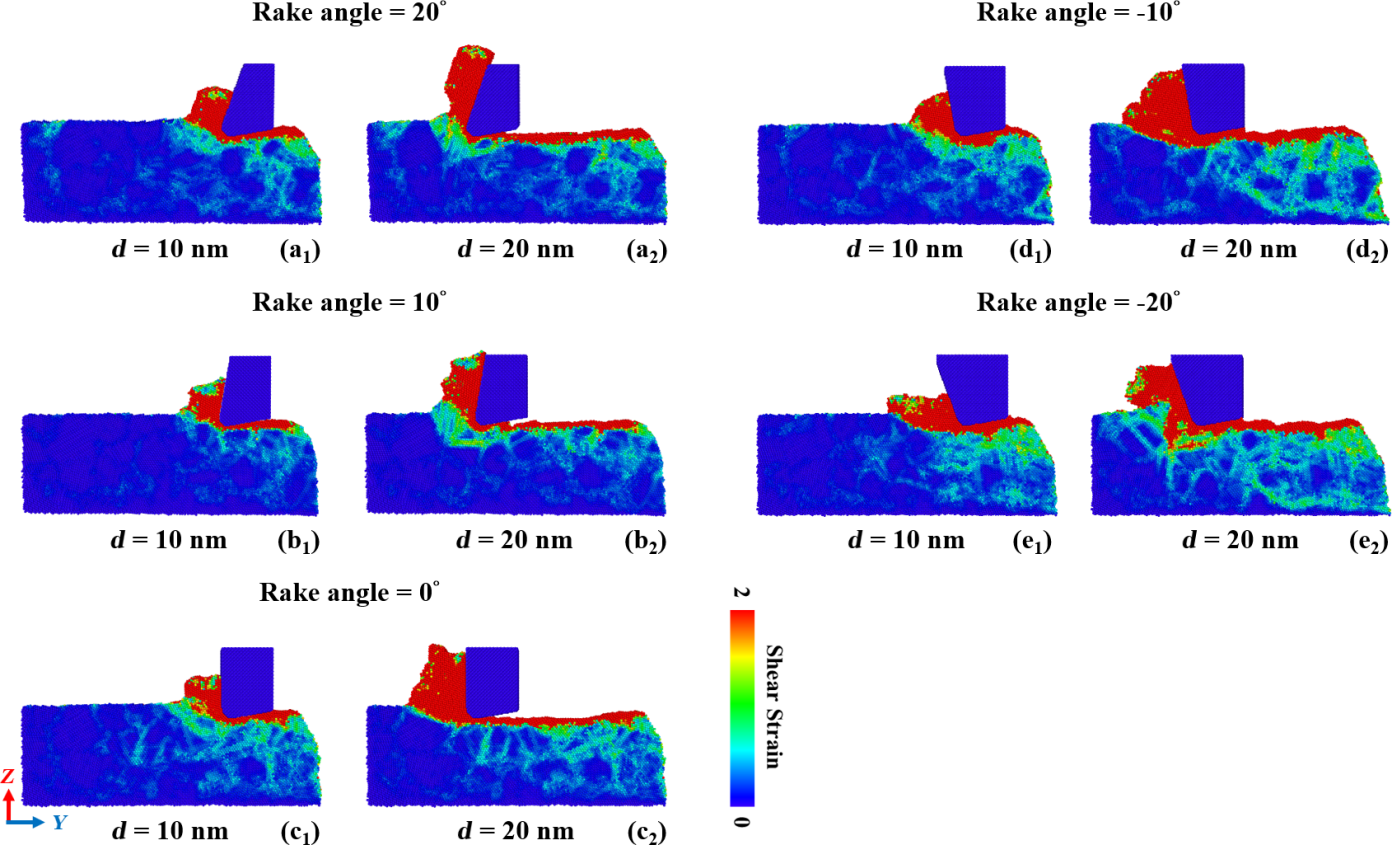
Shear strain distribution in GNG CoCrNi MEAs for various tool rake angles of (a) 20°, (b) 10°, (c) 0°, (d) −10°, and (e) −20°, with a cutting depth of 1 nm, a tool cutting-edge radius of 1 nm, a cutting speed of 10 m/s, and at a temperature of 300 K.

Figure 15 compares the shear strain distribution in GNG CoCrNi MEAs for various rake angles during the cutting process. The results show that, as the negative rake angle increases, the accumulation of material in the groove increases, and the chip volume decreases [44–45]. When the rake angle is 0° or −10°, the accumulation of material is concentrated at and attached to the tool’s front side. When the rake angle is −20°, it is more evident that the accumulation of material extends forward from the tool’s front. The material accumulation at a rake angle of −20° appears to move simultaneously forward and upward along the tool’s front side at a cutting length of 13 nm. As the rake angle increases, the chip size is almost the same for the positive tool rake angles. This means that a negative rake angles significantly affects the material removal rate, whereas positive rake angles have little impact on the material removal rate [45]. Moreover, for negative rake angles, the number of atoms under high shear strain increases, and the atoms forms shear bands, which are more likely to diffuse into the substrate.

**Figure 16:**
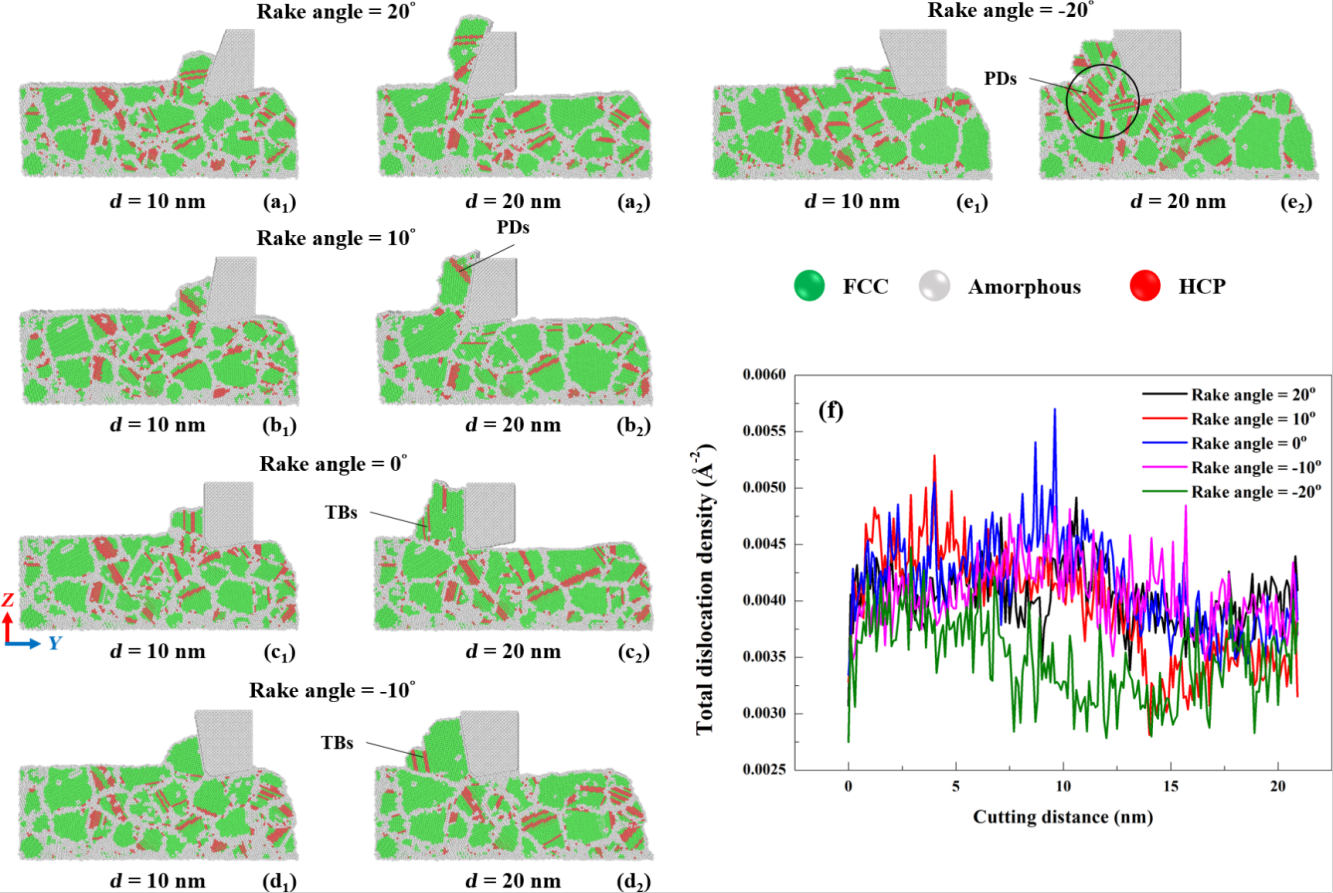
(a–e) Crystal structure evolution in CoCrNi MEAs for various tool rake angles and (f) the total dislocation density during cutting with a cutting depth of 1 nm, a tool cutting-edge radius of 1 nm, a cutting speed of 10 m/s, and at a temperature of 300 K.

Figure 16 displays the crystal structure evolution (Figure 16a–e) and the dislocation density (Figure 16f) of GNG CoCrNi MEAs for various rake angles during the cutting process. The crystal structure evolution results show that the mixed phase of FCC and HCP is maintained in the chip after cutting. The internal crystal structure undergoes a phase transformation from FCC to HCP during cutting. Stacking faults, twins, and grain boundary slips inside the grains affect the plastic deformation mechanism of polycrystalline CoCrNi MEAs [13]. The rake angle has little effect on the increase in amorphous phase atoms during the cutting process. However, according to the CNA results of the CoCrNi MEA substrates after cutting, substrate surfaces after cutting with a positive rake angle are flatter than those cut with a negative rake angle. A positive rake angle tool can result in less subsurface damage [45]. Regarding the dislocation density, Shockley and other dislocations mainly dominate the dislocation distribution produced in CoCrNi MEAs during cutting. Figure 14 shows that the tangential force of samples cut with negative rake angle increases with the decrease of thrust force, resulting in reduced compression force [43]. The cutting effect is enhanced, generating minor dislocations [44]. This explains why a rake angle of −20° yields the lowest dislocation density. Moreover, a rake angle of 10° yields a decreasing dislocation density as a function of the cutting length. For a rake angle of −20°, the dislocation density first decreases and then increases again; for all other rake angles, the dislocation density first increases and then decreases.

Table 4 compares the results for cutting forces, friction coefficients, and hardness values of some CoCrNi alloys from experimental works and MD simulations. In this work, the hardness was calculated using the following equation [46]:


[3] $$H=\frac{F_{\max}}{A_{\text{c}}}=\frac{F_{\max}}{\pi \left(2R-h\right)h},$$

where *F*_max_ and *A*_c_ are, respectively, the maximum indentation force and the contact area between the tool and the substrate measured at the nanoindentation depth, and *h* and *R* are the nanoindentation depth and tool radius, respectively. The results of this study were compared with the hardness results gathered through experiments [47–49]. In this study, the hardness of the polycrystalline CoCrNi MEAs is about 14.4 GPa, which is very different from the hardness of about 1.8 GPa gathered experimentally by Guo and coworkers [49]. The reason is the difference in the methods, such as the contrast between experiment and simulation, the size, the temperature, and the exactness of the data processing. The deformation mechanism of the material is very sensitive to the presence of defects [23].

**Table 4:** Comparison of cutting forces for comparable alloys.

Material	Resultant force (nN)	Friction coefficient	Hardness (GPa)	Method	Reference

polycrystalline CoCrFeMnNi HEAs	—	0.32–0.68	1.6	experiments	[47]
polycrystalline CoCrFeMnNi HEAs	51.2	—	1.8–1.9	experiments	[48]
NiCoCr MEAs	—	0.60–0.65	1.8	experiments	[49]
NiCoCr MPEAs	—	—	13.6	MD simulations	[50]
GNG CoCrNi MEAs	45.1–60.9	—	14.4	MD simulations	this study

For specimens in the range of 0.1–100 µm, the formation of voids at grain boundaries is the dominant defect and significantly affects the deformation behavior of the material. In contrast, voids seem negligible when the scale is changed to the nanoscale below 10 nm. Moreover, the simulation assumes perfect defects in polycrystalline CoCrNi MEAs, whereas the experimental samples always contain a variety of defects. However, the hardness results of the CoCrNi MEAs in this study are comparable to the MD results for NiCoCr multi-principal element alloys (MPEAs) from Akter et al. (13.6 GPa) [50]. Moreover, Table 4 shows that the resultant forces are similar to those obtained in a previous study [48].

## Conclusion

This study uses molecular dynamics to investigate the effects of grain size gradient structure, cutting depth, cutting-edge radius, and rake angle on CoCrNi MEAs during conventional cutting. The conclusions are: (1) The cutting chips transform into a mixed-phase structure of FCC and HCP. The plastic deformation at small average grain sizes is mainly dominated by grain boundary slip and grain rotation. The grains release energy as the machining proceeds and merge into larger grains. Grain boundaries do not affect partial dislocations when the grain size is large enough. (2) The average resultant force decreases with increased average grain size, which indicates a Hall–Petch relationship. Moreover, the cutting force increases with increased cutting depth, cutting-edge radius, and negative rake angle. (3) The stress is concentrated in the front of and below the cutting edge and transmitted through the grain boundaries. The shear strain diffusion area increases with the decrease in average grain size, increased cutting depth, cutting-edge radius, and negative rake angle. (4) Increased average grain sizes, decreased cutting depths, decreased cutting-edge radii, and positive rake angles of the tools can reduce the subsurface damage of the substrates.

## Data Availability

Additional research data is not shared.
